# Peptic ulcer disease complicated with choledocho-duodenal fistula and gastro-intestinal bleeding: a case report and review of the literature

**DOI:** 10.3389/fsurg.2023.1206828

**Published:** 2023-06-20

**Authors:** Isabelle Uhe, Alexis Litchinko, Emilie Liot

**Affiliations:** Division of Digestive Surgery, University Hospitals of Geneva, 1205 Genève, Switzerland

**Keywords:** gastro-intestinal bleeding, hematemesis, biliary fistula, choledochoduodenal fistula, helicobacter pylori

## Abstract

Peptic ulcer disease (PUD) is a very common condition, with an annual incidence ranging from 0.1% to 0.3% and a lifetime prevalence ranging from 5% to 10%. If not treated, it can lead to severe complications such as gastro-intestinal bleeding, perforation, or entero-biliary fistula. Entero-biliary fistulas and especially choledocho-duodenal fistula (CDF) are a rare, but relevant and important diagnosis, which can lead to several complications such as gastric outlet obstruction, bleeding, perforation, or recurrent cholangitis. In this article, we present the case of an 85-year-old woman with PUD complicated with gastro-intestinal bleeding and a CDF. We also performed a review of the literature to search for pre-existing cases with this atypical clinical presentation. The aim was to raise awareness among surgeons and clinicians by offering a summary of different types of entero-biliary and especially CDF, existing diagnostic investigations, and management.

## Introduction

Peptic ulcer disease (PUD) is a very common condition, with an annual incidence ranging from 0.1% to 0.3% and a lifetime prevalence ranging from 5% to 10% (1, 2). It is defined as a disruption of the mucosa which extends to the muscularis mucosa, and is usually caused by an Helicobacter pylori infection or the use of non-steroidal anti-inflammatory drugs (NSAID) (1, 2). Other reported risks factors are the use of corticosteroids, the presence of a tumour such as a gastric tumour or a lymphoma, stress, or a Zollinger-Ellison syndrome, caused by an increased acid secretion due to hypergastrinemia (1, 2). PUD may be located in the stomach or, more commonly, in the duodenum (1). Symptoms depend on the location of the ulcer, but patients usually report epigastric pain, fullness, nausea, loss of appetite, weight loss, hematemesis or melena (1, 3). If not treated, PUD can lead to severe complications such as gastro-intestinal bleeding causing haemorrhagic shock, perforation, cancer or entero-biliary fistula, all mostly requiring endoscopic, radiological or surgical treatment (1, 2).

Entero-biliary fistula is an abnormal communication between the biliary tract and the gastro-intestinal tract, and is due to gallstones in 90% of the cases (4, 5). It is a rare condition which can also result from PUD, tumours or Crohn disease involving the duodenum (4, 6). Different types of fistulas can be encountered, the most common being the cholecysto-duodenal fistula (68%–90%) (4, 7–9). There is also the cholecysto-colonic fistula (10%–13.6%), the choledocho-duodenal fistula (8.6%), the cholecysto-gastric fistula and the duodeno-left hepatic fistula, the last two being the rarest (4, 7–9). The diagnosis of entero-biliary fistula is challenging and often made incidentally during endoscopy or radiological imaging, especially because patients usually remain asymptomatic or report non-specific symptoms such as abdominal pain, nausea, fever, bowel obstruction or jaundice (4, 8).

In this article, we present the case of an 85-year-old woman admitted to the emergency department in haemorrhagic shock due to upper gastro-intestinal bleeding. PUD was diagnosed at endoscopy and a choledocho-duodenal fistula (CDF) was incidentally discovered during the examination. We performed a review of the literature to search for pre-existing cases with this atypical clinical presentation and compare the management of CDF.

## Materials and methods

We searched through MEDLINE and manual search for all existing articles reporting cases of CDF. The following search strategy was used: ((“Hematemesis”[MeSH Terms] OR “gastrointestinal bleeding”[Title/Abstract]) AND (“Biliary Fistula”[MeSH Terms]) OR “choledochoduodenal fistula”[Title/Abstract] OR “enterobiliary fistula”[Title/Abstract]) AND (“Case Reports”[Publication Type]). Only articles written in English or French about patients with a gastro-intestinal bleeding and a proven CDF were included. Duplicates, as well as articles reporting entero-biliary fistulas other than CDF, protocols, abstracts, meta-analysis, systematic reviews, retrospectives and prospective studies were excluded.

Two reviewers (IU, AL) independently selected the articles for inclusion and extracted the data. Discrepancies were solved by a third author (EL). The following data were extracted: country of the first author, date of publication, total number of patients, age and gender of the patient, symptomatology, method of diagnosis of the fistula and treatment.

## Results

### Case report

**Figure 1:**
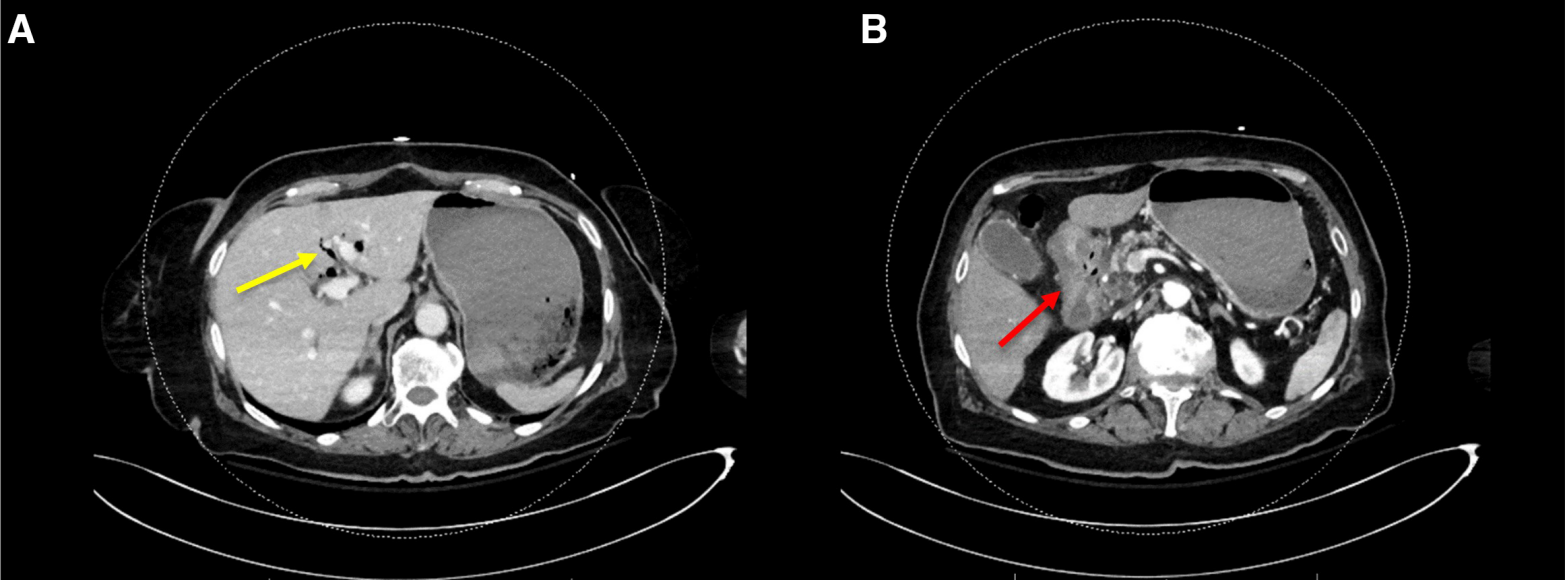
CT-scan of the abdomen showing (**A**) the presence of blood in the stomach, aerobilia (yellow arrow) and (**B**) inflammation of the first part of duodenum (red arrow).

**Figure 2:**
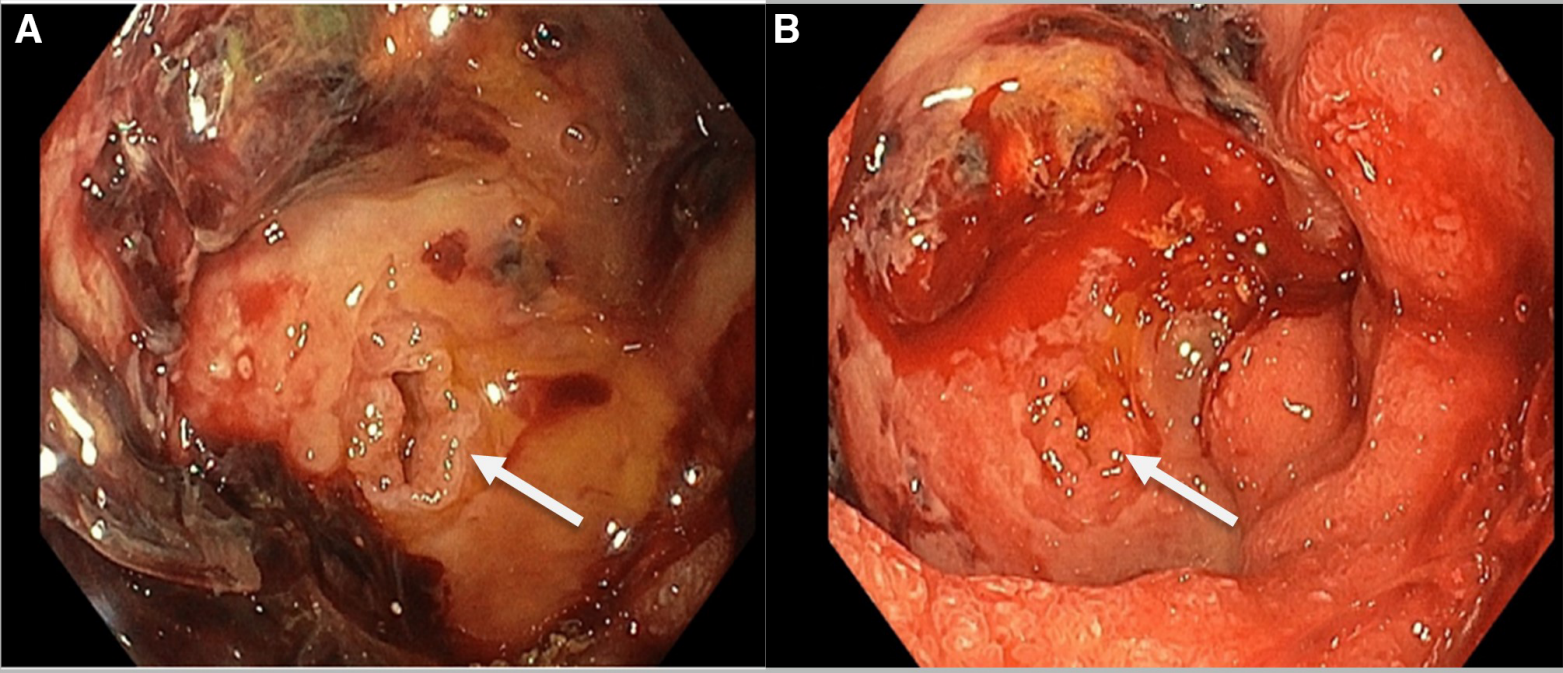
Endoscopic picture showing the 1A Forrest duodenal ulcer with the presence of a choledocho-duodenal fistula (CDF) and an active bleeding of the gastro-duodenal artery around the fistula (**B**).

**Figure 3:**
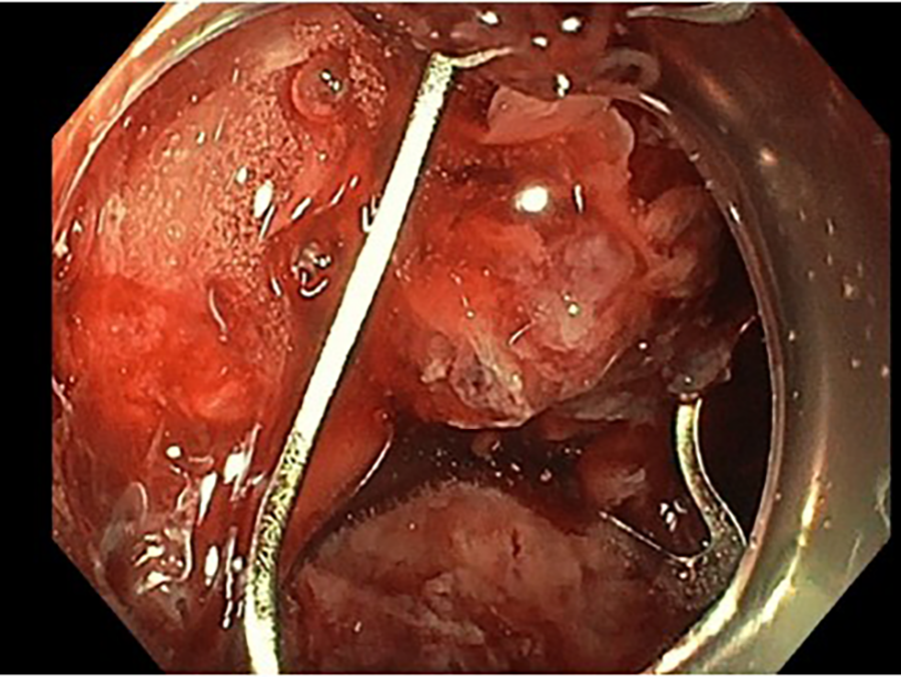
Endoscopic picture showing the 1A forrest duodenal ulcer treated with an over the scope clip.

An 85-year-old woman, known with hypertension and without any history of abdominal surgery, gallstones or allergies, was referred to the emergency department after having an episode of syncope associated with hematemesis and haematochezia. At admission, she showed signs of shock with diaphoresis, low blood pressure (57/42 mmHg), pulse rate 83 of beat/min and saturation of 100% under 2 L of oxygen. The medical history was limited due to acute confusion, but she reported epigastric pain for a week. She also mentioned a regular intake of NSAID for back pain. Physical examination revealed diffuse abdominal tenderness. Blood tests showed a metabolic acidosis (pH value of 7.27 and high lactate level of 5) and anaemia (haemoglobin of 77 g/l). After receiving 2 units of red blood cells, 1 litre of crystalloid solution and after initiation of amine support (Noradrenalin perfusion of 4.8 ml/h), her condition was stabilized, and a CT-scan of the abdomen was carried out. This revealed a CDF, inflammation of the first part of duodenum, signs of cholangitis and the presence of blood in the stomach (Figure 1). No gallstones were identified. A naso-gastric tube was placed, which removed 1.5 litre of blood from the stomach. An urgent esophagogastroduodenoscopy was performed, which showed a Forrest 1A posterior duodenal ulcer with an active bleeding of the gastro-duodenal artery, as well as the presence of air and bile, which was controlled with an Over The Scope Clip (Figures 2, 3). During the endoscopy, the patient developed low blood pressure which was treated with the administration of 1 litre of crystalloid solution, 20 g of albumin, 2 units of red blood cells and 2 g of fibrinogen. The patient was then transferred to the intensive care unit, where she remained stable. Noradrenalin pump was gradually withdrawn, and no further transfusion was needed. A Helicobacter pylori antibody test was carried out, which came out positive. She was therefore put under a treatment of proton pump inhibitors, as well as a course of three antibiotics for 14 days (Bismuth salts, metronidazole, and tetracycline). The patient was discharged 9 days after admission. At one month follow up, the outcome was still uneventful, and the patient was doing well. Since then, she has remained asymptomatic. No further treatment, especially surgical, has been performed.

### Literature review

**Figure 4:**
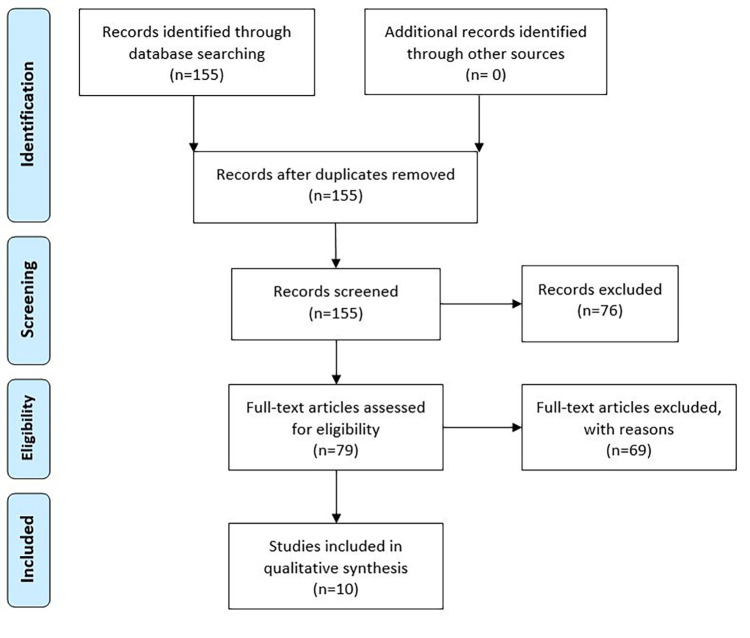
PRISMA flow diagram of literature search.

Regarding the literature search, 155 articles were identified from MEDLINE. No duplicates were removed. Forty-nine articles were excluded after title and abstract screening, and 27 articles were excluded because they were written in a foreign language, leaving 79 articles for full text analysis. Of those, five were excluded because patients presented with an entero-biliary fistula other than the CDF, 52 because patients were admitted in hospital with other symptoms than gastro-intestinal bleeding, and two were editorials. Ten articles were also excluded, because the full text could not be found. Finally, 10 articles were included (Figure 4 and Table 1).

**Table 1:** Summary of published case reports reporting choledocho-duodenal fistulas.

Author	Year	Country	DOI	Pts	Age	M/F	Symptoms	Investigations	Treatment of the CDF	Cause of CDF
Sreekumar S et al.	2021	UK	10.1136/bcr-2021-246532	1	75	M	Melaena and RUQ pain for 3 weeks	- CT: pneumobilia - Gastroscopy: anterio-medial ulcer at the D1/D2 junction, CDF	PPI, empiric H. Pylori eradication therapy	PUD
Yadav TN et al.	2020	Nepal	10.7759/cureus.11189	1	49	M	Melena for 7 days, syncopal attacks, generalized weakness	- Gastroscopy: multiple ulcers in the antrum, deformed pylorus - CT: distended stomach, distorted pylorus, thickened D1, pneumobilia involving the gallbladder and both the lobes of the liver - US: no gallstones	Duodenotomy, ligation of a posterior ulcer, cholecystectomy, Roux-en-Y choledochojejunostomy, posterior gastrojejunostomy, truncal vagotomy, jejunostomy	PUD
Misra D et al.	2020	USA	10.1177/23,24,709620934680	1	83	F	Fist consultation: Generalized abdominal pain for 4 days fever, altered mental status. Second consultation (7 months later): melena for 3 days Context: history of high-grade serous ovarian carcinoma, s/p surgery + chemotherapy, in recovery	Fist consultation: - CT: pneumobilia, gallstones, dilated intra- and extra HBD - MRI: similar findings as the CT - ERCP: duodenal ulcer superior to the major papilla, obstruction of the proximal CBD. Sphincterotomy + stent. Second consultation: - Gastroscopy: duodenal ulcer, fistulous tract. - MRI: fluid collection around the duodenum and pancreatic head with possible CDF. - Biopsy the fistula: poorly differentiated serous carcinoma	No treatment, palliative care due to multiples complications of cancer.	Metastatic ovarian carcinoma
Jacobs J et al.	2019	USA	10.1053/j.gastro.2019.03.053	1	85	F	Melena, epigastric pain, nausea and vomiting	- CT: pneumobilia - Gastroscopy: duodenal ulcer, CDF	PPI	PUD
Jiménez-Rosales R et al.	2018	Spain	10.1016/j.cgh.2017.10.013	1	84	F	Epigastric pain, hematemesis	- Gastroscopy: duodenal ulcer - CT: pneumobilia - 2dn gastroscopy: large ulcer with bile emanating from CDF	PPI	PUD
Antony A et al.	2016	USA	10.14309/crj.2016.17	1	76	F	Melena, abdominal pain, melena, coffee-ground emesis. Context: History of metastatic stage 4 colic adenocarcinoma, s/p chemotherapy + RHC	- Abdominal x-ray: pneumobilia - CT: mild biliary ductal dilatation, pneumobilia, progression of metastatic disease - Gastroscopy: severe narrowing of the duodenum, CDF distal to the narrowing.	Duodenal stent, palliative care	Metastatic colic adenocarcinoma
Mohite A et al.	2013	India	10.1016/j.jfma.2013.10.013	1	16	F	3 episodes of hematemesis, melena, LUQ abdominal pain for 6 months, fever and loss for 3 months.	- CT: chronic portal vein thrombosis with collaterals, pneumobilia, thickened CBD. - Gastroscopy: duodenal ulcer in the D1–D2 junction, D1 diverticulum, CDF. - Barium enema: CDF - Biopsy of duodenal ulcer: Mycobacterium tuberculosis	Anti-tubercular treatment	Tuberculosis
Chaudhari D et al.	2013	USA	10.1055/s-0032-1326266	1	56	M	Melena Context: patient with pancreatic cancer, treated with biliary metallic stent placement for obstructive jaundice	- CT: pneumobilia - Gastroscopy: duodenal ulceration around the stent, no bleeding. - Angio-CT: no active bleeding source	Patient died from exsanguination	Iatrogenic, due to metallic stent
Marsdin et al.	2011	UK	10.1136/bcr.05.2011.4275	1	88	M	Epigastric pain, coffee ground vomiting for 1 day	- Gastroscopy: blood in the stomach, large mass occluding the pylorus with suspicion of a gallstone, no ulcer. - CT: chronic cholecystitis, CDF involving D1 and the pylorus, impacted gallstone causing Bouveret's syndrome.	Subtotal cholecystectomy, oversew of the fistula, removal of the stone by gastrostomy	Gallstone
Naga M et al.	1991	Egypt	10.1055/s-2007-1010700	2	24	M	Hematemesis	- Gastroscopy: duodenal bleeding ulcer - Gastroscopy 2 months later: CDF	PPI	PUD
					70	F		- Gastroscopy: duodenal ulcer in the anterior wall of the bulb, CDF	PPI	

CDF, choledocho-duodenal fistula; ERCP, Endoscopic retrograde cholangiopancreatography; MRI, Magnetic resonance imaging; PPI, proton pump inhibitors; PUD, peptic ulcer disease; LUQ, left upper quadrant; RUQ, right upper quadrant; US, ultrasound.

### Articles and patient's characteristics

Among the 10 included case reports, four were written in the United States of America (10–13), three in Europe (14–16), one in Nepal (17), one in India (18) and one in Egypt (19) (Table 1). In total, 11 cases were reported. Most of the patients were female (6/11, 54.5%), with ages ranking from 16 to 88 years (mean age 64.2). All patients were admitted in hospital with clinical signs of upper gastro-intestinal bleeding, and five had also abdominal pain (5/11, 45.5%). Except for two patients, all of them had a CT scan, which revealed the presence of pneumobilia. A CDF was identified during gastroscopy in seven cases (7/11, 63.6%). For the other cases, the radiologic tools suggested the presence of the fistula.

Finally, six of the 11 cases of CDF were due to a PUD (6/11, 54.5%), two were related to a metastatic disease of colon and ovarian cancer (2/11, 18.2%), one was due to tuberculosis (1/11, 9.1%), one to gallstones (1/11, 9.1%) and one was iatrogenic (1/11, 9.1%) after metal stent placement in the common bile duct (Table 1).

Among the 11 patients with PUD, five received a conservative treatment with the administration of proton pump inhibitors. Only one patient required surgical treatment.

## Discussion

Peptic ulcer disease (PUD) is a common condition, which can lead to life threatening complications such as gastro-intestinal bleeding. It can also cause entero-biliary fistula, a rare but relevant and important diagnosis. The aim of this article was to highlight this subject, raise awareness among surgeons and clinicians and improve the management of this condition.

To diagnose PUD, the clinician should always get a detailed history and search for symptoms and signs such as epigastric pain, fullness sensation, intake of NSAID or other medication such as corticoids (1, 20). For patients younger than 50 years old without any red flags and if PUD is suspected, a conservative management with administration of a proton pump inhibitor, discontinuation of a possible NSAID treatment and/or eradication of Helicobacter pylori can be proposed (1, 20, 21). For patients older than 50 years old and in case of anaemia, gastro-intestinal bleeding, progressive dysphagia, tumour symptoms or failed medical treatment, esophagogastroduodenoscopy need to be performed (1, 21).

PUD is responsible for most upper gastro-intestinal bleedings (30%–60% of cases) (22–24). Esophagogastroduodenoscopy is an effective first line procedure, which allows to identify the source of haemorrhage and treat it (21, 25). When performed in the early setting (≤24 h of admission), it reduces significantly the rate of blood transfusion and the length of hospital stay (26). It also decreases the need for surgical procedure and mortality rate (27). In 1974, Dr John A.H. Forrest introduced a classification to unify the description of gastro-intestinal bleeding and stratified the risk of recurrent bleeding and the mortality rate (28). The “Forrest classification” includes 3 stages; an active bleeding is present in stage 1 (1a spurting bleeding, 1b oozing bleeding), a recent bleeding in stage 2 (2a visible non-bleeding vessel, 2b adherent clot, 2c hematin covered ulcer) and stage 3 is characterized by the presence of a lesion without signs of haemorrhage (29, 30). Stages 1a to 2a require an endoscopic haemostasis, which can be achieved by several modalities such as epinephrine injection, hemospray or Over The Scope Clip, as in the situation described in this article (25). Endoscopy remains also the treatment of choice in case of recurrent bleeding (25). If the procedure fails, angiography with arterial embolization can be proposed (25). Surgical treatment is also an option, especially in case of hemodynamic instability and/or in the presence of an ulcer larger than 2 cm (25). An open approach, appropriate to location and size of the ulcer, should therefore be preferred (25). Interestingly, the duodenal location is associated with higher reoperation rate and 90 days mortality rate (25, 31).

In the case we reported, the patient did not only have a gastro-intestinal bleeding due to PUD, but also a choledocho-duodenal fistula (CDF), which increases the complexity of the management, especially if a surgical procedure is required.

Most of entero-biliary fistulas and especially cholecysto-duodenal fistulas are caused by the present of gallstones, which induce inflammation, necrosis of the gallbladder or of the common bile duct wall and fistulisation into the adjacent organ (4, 5, 7, 32). PUD of the duodenum is responsible for only 5%–6% of entero-biliary fistulas, but 75%–80% of CDF (32, 33). In our review, 54.5% of CDF cases were due to PUD. Two types of CDF are described in the literature: the proximal and the distal type (7, 34). The proximal type is a single fistula, located more than 2 cm above the papilla and drains into the posterior wall of the duodenum (7, 34). This is usually caused by PUD, but diverticulitis of the duodenum, iatrogenic causes (for instance after laparoscopic cholecystectomy or retrograde cholangio-pancreatogram ERCP), or carcinoma have also been described (7, 35, 36). A carcinoma (for example a cholangio-carcinoma) or a metastasis can lead to a fistula by compressing the common bile duct, therefore increasing the pressure and weakening the duct wall, or by infiltrating the tissues and causing a necrosis (5, 10, 35). In our review, two cases of CDF were related to a metastatic disease of colon and ovarian cancer. Patients with a proximal fistula often complain about dyspepsia, but they can also have gastro-intestinal bleeding, which can have a significant impact if the gastro-duodenal artery is involved, as in this case report (7). The distal type, also called peri-papillar type, can be a single or multiple fistula and is located within 2 cm of the distal common bile duct (7, 34, 37). This is commonly caused by gallstones, eroding the common bile duct and creating a fistula (7).

**Figure 5:**
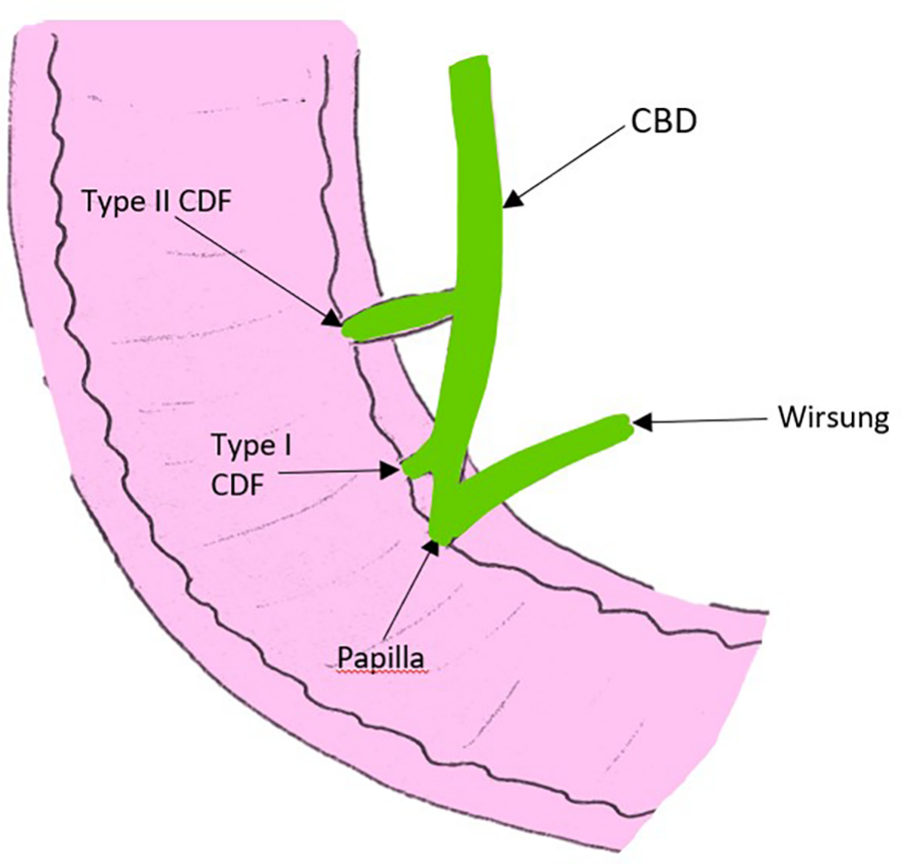
Picture adapted from the article of Ikeda et al., illustrating the two types of CDF according to the location of the fistula; the type I located in the intra-mural portion of the common bile duct right next to the papilla, and the type II located in the extra-mural portion of the common bile duct. CBD, common bile duct; CDF, choledocho-duodenal fistula.

In 1975, Ikeda et al. also proposed a classification of CDF according to the location of the fistula; the type I and II, as illustrated in Figure 5 (38). The type I is located in the longitudinal fold right next to the papilla (the intra-mural portion of the common bile duct), and results from the impaction of a gallstone not small enough to pass through the papilla (38). The type II forms in the extra-mural portion of the common bile duct and is due to larger stones (38).

Diagnosis of entero-biliary fistula is made by endoscopy and ERCP, which not only allow to visualize the fistula orifice and tract trough the injection of contrast, but also to diagnose the aetiology and sometimes treat it (4, 7, 39). Computed tomography (CT) is another valuable diagnostic tool, which can reveal the presence of air in the biliary tree (4, 32, 37). According to Shimono et al., it helps to differentiate a gallbladder-enteric fistula from a choledocho-enteric fistula (40). Plain abdominal radiography can also demonstrate air in the biliary tree, but only 5%–10% of the time (40).

Several therapies have been proposed to treat entero-biliary fistula but no guidelines have been established yet (6, 8). It is recommended to treat cholecysto-duodenal fistula surgically with cholecystectomy and closure of the fistula tract (40–42). CDF incidentally diagnosed and caused by PUD could be managed conservatively, with the administration of proton pump inhibitor, as mentioned in our article (5, 32, 39, 43). Our review also showed that most of the cases were managed conservatively. If the fistula is caused by a gallstone and is located distally, ERCP with sphincterotomy can be performed, followed by a cholecystectomy (6, 7, 39). Zhao et al. reported a case where CDF was successfully treated with the implantation of a covered self-expandable metal stent in the common bile duct (44). Neumann et al. also published a case similar to the one we described, where a CDF caused by a PUD was totally closed after using an Over The Scope Clip (45).

According to several authors, a surgical approach should be advised in case of failed medical treatment, gastric outlet obstruction, haemorrhage, perforation, recurrent gallstone ileus or recurrent cholangitis (5, 8, 32, 43). For CDF due to PUD refractory to medical treatment, a vagotomy with an antrectomy and a reconstruction according to Billroth II procedure with a gastrojejunostomy can be performed (7, 32, 33, 39). To note, the fistula should in this case be left intact (7, 32, 33, 39). To determine whether the size of the CDF has an impact on symptoms and treatments, Li et al. reviewed 50 cases during a 14-year period (46). They concluded that large fistulas increase the risk of cholangitis and proposed therefore the following strategies. For orifices larger than 1 cm and common bile duct larger than 2 cm, associated with complications in the biliary system such as strictures of an intrahepatic duct, patients should undergo surgical treatment (for instance Roux-en-Y intrahepatic cholangio-jejunostomy, Roux-en-Y choledocho-jejunostomy or partial liver resection) (46). For orifices between 0.5 and 1 cm with a common bile duct dilated over 2 cm, without complications in the biliary system, a side-to-side choledocho-jejunostomy without a transection of the duct can be achieved (46). For orifices larger than 0.5 cm with a common bile duct dilated over 1.2 cm and caused by a gallstone, a stone removal followed by a cholecystectomy should be performed (46). Finally, for orifices smaller than 0.5 cm, a conservative management can be applied (46).

Through this manuscript, we provide the information available for the reader to recognize, diagnose and manage cases of entero-biliary fistula and especially CDF. We also conducted a review of the literature to search for pre-existing cases of CDF manifesting as gastrointestinal bleeding, offering therefore the first comprehensive review on the subject. The pictures are of good quality, adding a lot of value to this manuscript, compared with other published articles.

## Conclusion

Choledocho-duodenal fistulas are a rare condition, often diagnosed incidentally during endoscopy or radiological imaging. Most of the patients remain asymptomatic or report non-specific symptoms such as abdominal pain, fever, or bowel obstruction. To date, no clear guidelines to treat entero-biliary fistula have been established yet, but most of choledocho-duodenal fistulas caused by peptic ulcer disease can be managed conservatively, with the administration of proton pump inhibitor and the eradication of Helicobacter Pylori infection. Endoscopic treatment including ERCP with sphincterotomy, the implantation of a covered self-expandable metal stent in the common bile duct of the use of an Over The Scope Clip can also be proposed. A surgical approach should be advised in case of failed medical treatment or complications, such as gastric outlet obstruction or recurrent cholangitis.
